# The Dark Side of Antioxidants: When Scavenging ROS Undermines Plant Stress Acclimation

**DOI:** 10.3390/antiox15080965

**Published:** 2026-08-02

**Authors:** Panqi Qiu, Ziwei Chu, Yurong Xie

**Affiliations:** 1 Beijing Key Laboratory of Maize Bio-Breeding, Research Institute of Biology and Agriculture, School of Advanced Agricultural Sciences, University of Science and Technology Beijing, Beijing 100083, China; d202510947@xs.ustb.edu.cn (P.Q.); m202511515@xs.ustb.edu.cn (Z.C.); 2 Beijing Engineering Laboratory of Main Crop Bio-Tech Breeding, Beijing International Science and Technology Cooperation Base of Bio-Tech Breeding, Zhongzhi International Institute of Agricultural Biosciences, Beijing 100083, China

**Keywords:** redox homeostasis, ROS signaling, growth–defense resource trade-off, plant stress acclimation

## Abstract

Reactive oxygen species (ROS) exert dual biological functions in plants. Though they form toxic byproducts of aerobic metabolism, ROS also serve as indispensable secondary messengers that orchestrate stress acclimation programs. For decades, plant physiologists operated under a pervasive assumption that constitutive and non-compartmentalized upregulation of antioxidant capacity would universally enhance abiotic stress tolerance. This long-standing dogma has now been thoroughly overturned. A growing body of evidence shows that sustained, global high antioxidant activity often impairs adaptation rather than helping it. In this review, we replace the simplistic “more antioxidants equal better tolerance” framework with a dynamic model of cellular redox homeostasis. We dissect three interconnected mechanisms though which unrestrained ROS scavenging generates deleterious phenotypic outcomes. First, indiscriminate clearance blunts transient ROS pulses and propagating ROS waves, the core signaling events acquired to trigger systemic acquired acclimation (SAA). Second, continuous antioxidant biosynthesis drains finite carbon skeletons, NADPH, and ATP pools, exacerbating evolutionary growth-defense resource trade-offs. Third, non-specific bulk ROS scavenging erases compartment-specific organellar retrograde signals, which rely on tightly controlled spatial and temporal ROS fluctuations. We concurrently define physiological boundary conditions where robust antioxidant activity remains vital for plant survival under extreme stress. Rather than advocating for the complete suppression of ROS detoxification, our analysis advocates context-dependent fine-tuning of redox signaling networks. We also summarize emerging precision redox monitoring and genetic engineering tools, and outline translational breeding pipelines to develop climate-resilient crops that balance stress survival and yield stability. This work delivers novel conceptual perspectives to advance fundamental plant redox biology.

## 1. Introduction

Reactive oxygen species (ROS) exhibit inherently bifunctional roles in plant stress biology. Beyond their reputation as toxic byproducts of aerobic metabolism, ROS act as essential secondary messengers that orchestrate local and systemic signaling for stress acclimation [1,2,3,4]. This duality has long hampered progress in plant stress physiology, prompting a conceptual evolution from static antioxidant maximization toward dynamic redox fine-tuning [5,6]. The field’s evolution can be partitioned into distinct paradigms, including an initial damage-centric paradigm, a subsequent phase recognizing ROS as signaling molecules, and the current framework emphasizing spatiotemporal redox dynamics [7,8,9,10]. Early work in the first phase treated ROS solely as damaging agents, establishing a linear dogma: stress disrupts redox balance and triggers ROS overaccumulation, which damages macromolecules. This dogma led to the assumption that augmented antioxidant defense directly enhances tolerance [11,12,13,14]. This model persisted in part because key antioxidant enzymes are readily quantifiable, serving as ubiquitous surrogate biomarkers for stress resilience [15,16,17,18].

Accumulating evidence now challenges key aspects of this damage-defense linearity. First, numerous studies document unaltered or even repressed antioxidant systems under stress [12,19]. Additionally, ROS are recognized as bona fide signals driving transcriptional reprogramming via propagating systemic “ROS waves” [20,21,22,23,24,25,26,27]. In many contexts, constitutively elevated antioxidant activity attenuates stress-dependent ROS signaling and compromises resistance [9,12,23,28,29,30,31,32]. Second, sustained high antioxidant pools impose steep metabolic costs, including consuming carbon skeletons, reducing equivalents, and ATP, which exacerbates growth–defense trade-offs that reduce fitness under resource limitation [33,34]. If constitutive hyperactive antioxidant machinery were truly advantageous, natural selection would have fixed it across plant lineages. Its absence in most wild accessions under non-stress conditions therefore reveals inherent fitness costs.

A critical analysis by Xu et al. [12] first challenged the reliability of antioxidant enzyme activity as a proxy for abiotic stress tolerance, demonstrating that constitutively elevated antioxidant machinery attenuates stress-dependent ROS-mediated stress signaling and impairs acclimation. Earlier reviews have thoroughly covered the protective roles of antioxidants and the signaling functions of ROS, but few have asked what happens when antioxidant activity becomes excessive. The present review takes up this question. We expand Xu and colleagues’ methodological critique into a broader conceptual reassessment, focusing on three specific issues. How does non-selective scavenging disrupt compartment-specific and time resolved ROS signaling? What metabolic costs drive constitutive antioxidant overexpression? And when is antioxidant defense indispensable versus when does it backfire? We also outline strategies for redox-targeted crop breeding. Our argument is built on three interconnected pillars. First, ROS operate primarily as signals that trigger acclimation, and indiscriminate scavenging blunts those signals. Second, constitutively high antioxidant levels carry metabolic costs that create growth–defense resource trade-offs. Third, ROS signaling relies on tight spatiotemporal control, so blanket elimination does more harm than good. We also specify the conditions under which antioxidants remain essential, to keep the argument from becoming one-sided.

**Figure 1:**
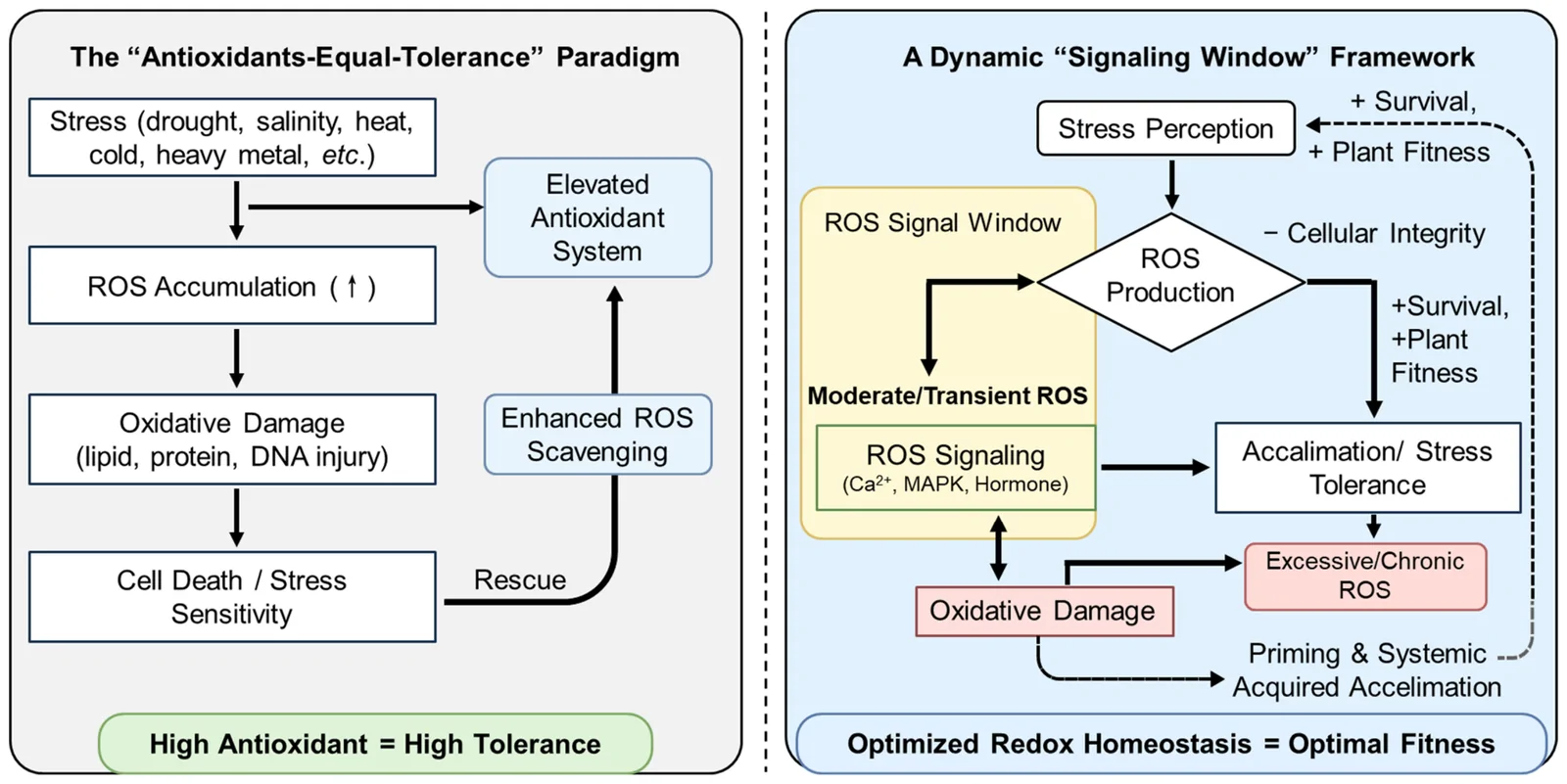
Schematic comparison of the conventional linear antioxidant-tolerance paradigm and the novel dynamic ROS signaling window framework. (**Left panel**): The traditional unidirectional model assumes that excessive ROS accumulation drives irreversible oxidative damage and cell death, while enhanced antioxidant activity simply scavenges ROS to rescue stress sensitivity, leading to the binary conclusion that higher antioxidant capacity equals stronger stress tolerance. (**Right panel**): The proposed dynamic framework divides ROS into two functional pools dependent on concentration and duration. Moderate transient ROS acts as essential signal triggers for stress acclimation (green zone), whereas persistent high ROS causes cellular injury (red zone). Antioxidants function as fine-tuners to maintain ROS within a permissive signaling window rather than eliminating ROS entirely. A feedback loop from established acclimation back to stress perception illustrates stress priming and SAA. This conceptual framework is developed in the following sections and underpins the precision redox engineering strategies proposed later in this review. This figure presents a conceptual model synthesized from the literature reviewed and does not represent empirical data from a single experiment.

Importantly, we are not dismissing antioxidant research. That field has generated foundational insights into plant stress physiology. Instead, we are trying to push it in a different direction. Rather than relying on descriptive measurements of antioxidant activity, we aim for precision redox engineering. We want to replace static endpoint assays with dynamic in vivo imaging. Instead of overexpressing single genes, we propose network-level tuning. Earlier reviews have tended to treat ROS signaling and antioxidant defense as separate topics. Our review brings them together to ask where the line falls between beneficial and detrimental antioxidant activity. This distinction matters for both basic research and crop engineering. Thus our goal is not to reject antioxidants outright, but to shift the objective from maximizing scavenging to optimizing redox homeostasis in a dynamic and context-dependent way (Figure 1).

The literature covered in this review was identified through searches in PubMed, Web of Science, and Google Scholar using combinations of the following keywords: “antioxidant enzymes”, “ROS signaling”, “plant stress tolerance”, “growth–defense trade-off”, “oxidative stress”, “systemic acquired acclimation”, and “redox regulation”. We prioritized peer-reviewed articles published between 2000 and 2026, with emphasis on studies from the past decade, while also including foundational earlier work where relevant to the conceptual framework. The selection of cited studies was guided by their contribution to the mechanistic understanding of ROS–antioxidant interactions, rather than by exhaustive enumeration of all available literature. This is a narrative review that synthesizes and interprets the literature to propose a conceptual framework for precision redox regulation in plants, rather than a systematic meta-analysis.

We conclude by outlining forward-looking research directions, including redox-sensitive post-translational modifications, live-cell ROS imaging, and network-level redox engineering. These directions can guide the development of climate-resilient crops that balance survival and productivity [9,35].

## 2. The Prevalence of Paradoxes: When Antioxidants Fail to Protect

The long-standing assumption that amplified antioxidant capacity directly improves stress tolerance relies on a deceptively simple chain of reasoning. ROS accumulation triggers cellular damage. Antioxidants neutralize ROS. Therefore, more antioxidants should minimize injury and strengthen resilience [3,36,37,38]. However, this neat logic does not survive contact with the data. Accumulated empirical observations contradict it, revealing paradoxical phenomena across diverse species and stress regimes (Table 1). Numerous independent studies have documented unchanged or even suppressed antioxidant enzyme activity in stressed plant tissues. These findings fundamentally undermine the core premise that antioxidant upregulation is a mandatory adaptive response to environmental adversity [12,39,40,41].

Several representative cases deserve a closer look. Catalase (CAT) is one of the most widely quantified antioxidant enzymes, and it exhibits consistent downregulation under multiple abiotic stressors. Under heat stress, CAT activity declined in a broad range of species [51]. Under drought, similar suppression occurs in both herbaceous (*Salvia miltiorrhiza, Helianthus annuus*) and woody species (oak and pine) [46,47,48]. Even under chilling and high irradiance, CAT repression is observed alongside differential regulation of other antioxidant enzymes such as SOD, APX, and GR, which are simultaneously upregulated in cucumber under chilling [52,53]. If antioxidant enzymes were a primary determinant of stress resistance, their activity would uniformly rise upon stress exposure. However, the published datasets show otherwise [12,28,45]. In barley, for instance, antioxidant activities were constitutively higher in a salt-sensitive variety than in a tolerant one, and SOD activity showed no correlation with salinity tolerance [49]. In Arabidopsis, four natural accessions showed no correlation between freezing tolerance and antioxidant enzyme activity [45]. Catalase activity dropped across a range of chilling-sensitive and -tolerant species [50]. In soybean nodules exposed to cadmium, the antioxidant defense system showed no measurable response [54].

Genetic manipulation experiments offer even more compelling evidence against the “more antioxidants equal better tolerance” view. Transgenic tobacco constitutively overexpressing GST/GPX showed no improvement in paraquat or photooxidative stress tolerance [42,43]. More recently, overexpression of maize *ZmGST26* in Arabidopsis decreased rather than improved drought resistance, with transgenic lines exhibiting reduced CAT, SOD, and POD activities alongside increased ROS accumulation [30]. More strikingly, in citrus, stable transgenic lines overexpressing phospholipid hydroperoxide glutathione peroxidase (PHGPx) could only be regenerated after mutating the enzyme’s catalytic active site to abolish its ROS-scavenging function, demonstrating that excessive PHGPx activity disrupts ROS-dependent shoot organogenesis [29]. In the metal hyperaccumulator *Sedum alfredii*, heterologous overexpression of cyanobacterial Fe-SOD (NfFeSOD) led to severe growth retardation and distorted root architecture, with ~50% reduction in cellular H_2_O_2_ and global attenuation of ROS-responsive transcriptional cascades [44]. Additional transcriptional evidence derives from poplar lines overexpressing PtoMYB99, which suppresses SOD, POD, and CAT activity while elevating the ROS and MDA levels, ultimately weakening osmotic stress tolerance [31]. In maize, overexpression of *ZmWRKY74* similarly reduced drought tolerance by suppressing antioxidant enzyme activities and proline accumulation [32].

Intraspecific natural variation studies provide orthogonal validation of this paradox. A large-scale screening in barley revealed that salt-sensitive genotypes maintained significantly higher constitutive antioxidant enzyme activity than salt-tolerant counterparts, with no detectable positive correlation between SOD activity and salinity tolerance [28]. This finding has been corroborated by recent studies highlighting genotype-specific antioxidant responses to salinity stress [55]. Similarly, four distinct *Arabidopsis thaliana* natural accessions exhibited no correlation between freezing tolerance and basal antioxidant enzyme activity, arguing against constitutive antioxidant abundance as a reliable predictor of cold acclimation capacity [45]. This conclusion is supported by recent transcriptomic and metabolomic findings that emphasize the complexity of freezing tolerance mechanisms beyond simple antioxidant capacity enhancement [56,57,58]. Synthesizing these transgenic and germplasm datasets, Xu et al. [12] formally concluded that constitutively elevated antioxidant activity interferes with stress-triggered ROS signaling and impairs plant acclimation, questioning the utility of antioxidant enzyme activity as a universal biomarker or breeding target for abiotic stress resilience.

From an evolutionary perspective, these paradoxical observations become even more striking. If persistently high antioxidant capacity conferred universal fitness advantages, natural selection would have favored plant genotypes with constitutively activated antioxidant systems across all habitats. However, ascorbate, a major antioxidant, has been subject to selection pressures during evolution, with its accumulation levels determined by cost–benefit resource trade-offs rather than maximization [59]. The near-universal transcriptional and post-translational suppression of antioxidant machinery under non-stress conditions implies strong evolutionary constraints. Constant investment in high antioxidant levels imposes fitness costs that outweigh potential protective benefits in most environments [12]. Indeed, crop domestication frequently reduces fruit antioxidant content through artificial selection for accelerated vegetative growth and higher yield, evidencing a metabolic resource trade-off between nutritional optimization and stress resilience [60]. In natural ecosystems, plants coordinate growth–defense resource trade-offs through evolutionary-optimized regulatory circuits, rather than maximizing defense at all times [61]. Induced defenses are considered less expensive than constitutive preformed defenses, because costs are realized only when required. This explains why plants do not maintain constitutively high antioxidant levels across all habitats [62,63].

The observations catalogued above, unchanged or suppressed antioxidant activity under stress, failed transgenic complementation, and the absence of positive correlation across natural accessions, collectively point to a common underlying explanation. ROS are not merely toxic byproducts to be eliminated; but primary signaling molecules whose controlled accumulation is essential for stress acclimation [12,64]. If antioxidants were simply defense weapons against oxidative damage, their stress-induced upregulation would be uniformly observed. The fact that they are frequently downregulated suggests that plants actively modulate antioxidant capacity to preserve ROS signaling functions [64].

**Figure 2:**
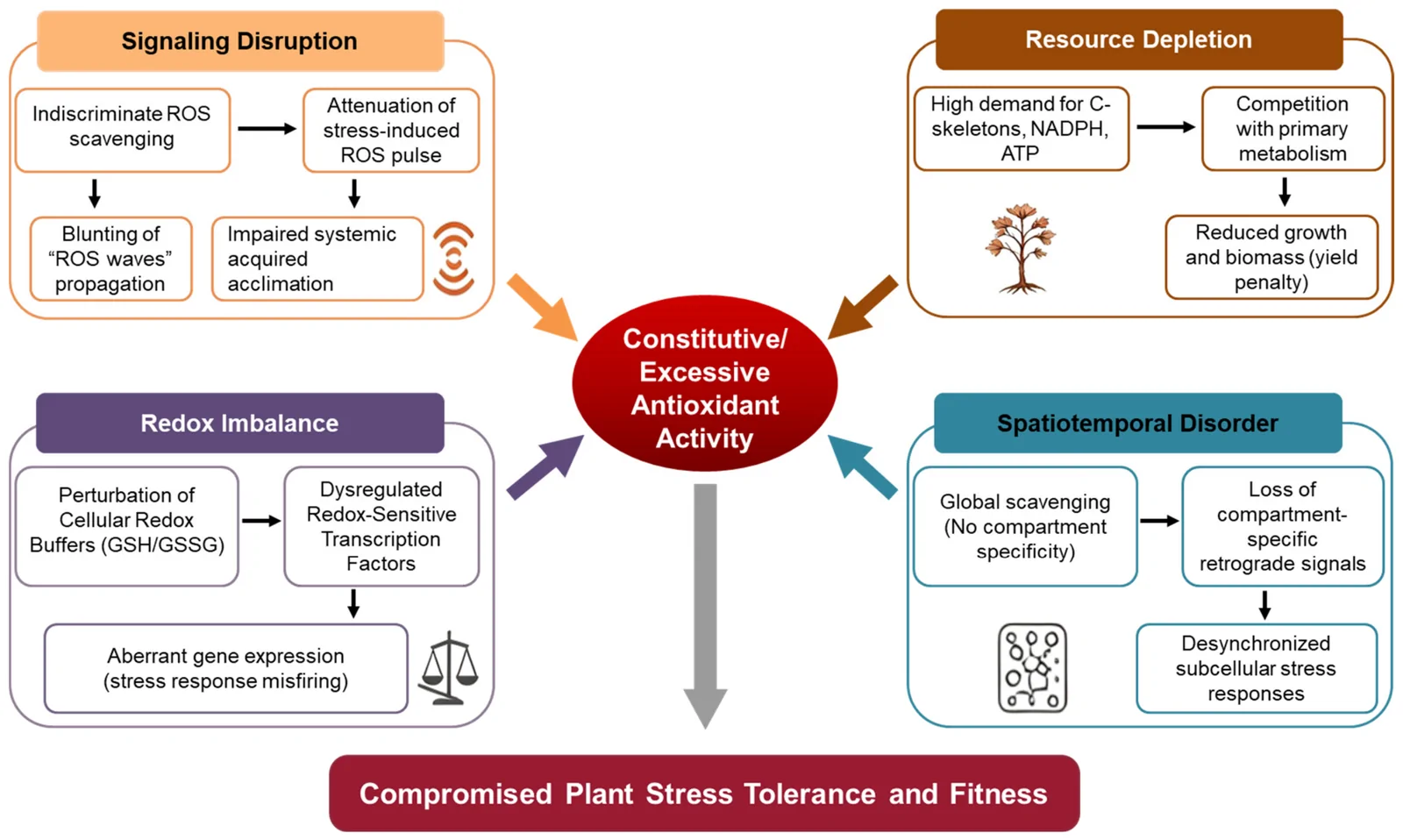
Hub-and-spoke schematic illustrating four core mechanisms through which constitutive or excessively elevated antioxidant activity negatively suppresses plant stress acclimation. At the diagram center, the hub node represents persistent high antioxidant capacity, defined as the “dark side” of antioxidant systems under stress. Four distinct colored branches radiate outward to outline sequential adverse cascades: (1) The orange upper-left branch describes indiscriminate ROS elimination dampens stress-triggered ROS pulses and blocks long-distance ROS wave transmission, leading to defective SAA; (2) The tan upper-right branch shows sustained antioxidant biosynthesis consumes carbon skeletons, NADPH, and ATP, competing with primary metabolic pathways and causing growth retardation and yield losses; (3) The purple lower-left branch depicts downstream consequences of disrupted redox homeostasis (e.g., GSH/GSSG ratios) that further misregulate redox-sensitive transcription factors, representing a secondary amplification of the primary mechanisms described above; (4) The teal lower-right branch highlights non-compartment-specific global ROS scavenging abolishes organelle retrograde signaling, desynchronizing subcellular stress response programs. All four detrimental phenotypic outcomes converge to the bottom terminal banner, summarizing the ultimate consequence: overall weakened stress tolerance and reduced plant fitness under adverse environments. Small linear warning icons adjacent to each terminal phenotype box visually mark the harmful physiological outcomes triggered by overactive antioxidant systems. This schematic is a conceptual model based on the literature reviewed, illustrating the mechanistic framework proposed in this review.

These four adverse outcomes, summarized in Figure 2, provide the conceptual roadmap for the mechanistic dissections that follow. Branches (1)–(4) respectively correspond to signal interference, growth–defense resource trade-offs, downstream redox misregulation, and spatiotemporal disruption, each of which is examined in turn in the following sections.

## 3. ROS Are Signals First, Toxins Second

To resolve the widespread paradox outlined above, we must revisit the dual biological identity of ROS. The long-standing dogma framing ROS merely as unavoidable toxic byproducts of aerobic metabolism has undergone fundamental revision over the past two decades [9,64]. ROS are now recognized as ubiquitous, context-dependent signaling hubs that integrate developmental, metabolic, and stress cues to shape adaptive plant phenotypes, linking central energy metabolism to growth homeostasis and environmental resilience [3,65,66,67]. At basal physiological levels within chloroplasts, mitochondria, and peroxisomes, ROS operate as indispensable secondary messengers governing stomatal dynamics, programmed cell death (PCD), and SAA, rather than functioning solely as cellular damaging agents [3]. This signaling function is not a secondary side effect of ROS production; it constitutes their core physiological role in plant environmental sensing [68].

### 3.1. ROS as Secondary Messengers Coordinating Plant Growth, Development and Stress Responses

Under homeostatic conditions, low-amplitude ROS bursts orchestrate core plant developmental programs by modulating the activity of transcription factors such as the WRKY, NAC, and ZAT families, which regulates genes involved in root hair elongation, xylem differentiation, and flowering time [69]. Controlled ROS concentrations activate conserved defense cascades upon stress exposure, acting as molecular switches that remodel genome-wide transcription through the oxidative modification of cysteine residues in key signaling proteins such as NPR1 and TGA transcription factors [70,71,72]. Upon environmental challenge, ROS trigger cascades of transcription factor activation, inducing hundreds of stress-responsive genes required for acclimation [69]. Together with reactive nitrogen species (RNS), ROS maintain a tightly calibrated cellular redox rheostat that balances signal transduction and oxidative damage avoidance [4].

ROS signaling forms an interconnected regulatory network with calcium (Ca^2+^) waves, two core local signal modules that coordinate stress responses via shared intermediate protein nodes such as calmodulin and CDPKs [73]. ROS further interact synergistically with phytohormones, including abscisic acid (ABA), salicylic acid (SA), jasmonic acid (JA), ethylene, and brassinosteroids, to tailor stress-specific physiological outputs [74,75]. NADPH oxidase-derived ROS function as upstream triggers of temperature stress hormone signaling, initiating feedback regulatory loops that establish stress acclimation [76]. These local signaling events collectively form the building blocks for the systemic responses discussed below.

### 3.2. The ROS Wave and Systemic Acquired Acclimation

The most definitive evidence supporting ROS as primary stress signals originates from characterization of the ROS wave, a cell-to-cell propagating signal moving at 8 cm min^−1^ that mediates plant responses to light, heat, salinity, mechanical wounding, and pathogen attack [27]. The ROS wave initiates and sustains SAA, an essential whole-plant adaptive response that preconditions distal unexposed tissues to withstand subsequent stress episodes [25,27,77]. During propagation, ROS waves dynamically crosstalk with calcium fluxes, electrical signals, and phytohormone gradients without rigid hierarchical control, forming a flexible signal network that coordinates multi-tissue stress responses [73]. Notably, systemically propagating Ca^2+^ and ROS waves function together with electric signals in directional cell-to-cell systemic signaling [73]. Among the different systemic signals triggered by stress in plants are electric, calcium, ROS, and redox waves that are mobilized in a cell-to-cell fashion from local to systemic tissues over long distances, sometimes at speeds of up to several millimeters per second [78].

The core molecular machinery sustaining ROS wave transmission relies on respiratory burst oxidase homolog D (RBOHD), which auto-amplifies ROS signals along vascular bundles to reach distant leaf and root tissues [79,80]. In this process, apoplastic ROS initiates long-distance signaling. Each cell in the path can activate its own ROS production via the activation of RBOHD, termed the ROS wave [79]. RBOHD and RBOHF are both required for local and systemic ROS signaling at the vascular bundles of Arabidopsis [80]. Moreover, RBOHD-generated ROS orchestrate cell-to-cell spread of systemic signals through plasmodesmata [80]. Once perceived by mesophyll cells in unstressed organs, ROS signals trigger tissue-localized defense reprogramming. Organic pollutants can trigger long-distance ROS translocation from leaves to roots via a Ca^2+^–RBOH–ROS module, further recruiting beneficial rhizosphere microbiota to reinforce systemic tolerance [81]. These observations collectively establish ROS waves as a foundational mechanism for whole-plant stress integration.

### 3.3. Critical Physiological Consequences for Antioxidant Research

The discussion above establishes that ROS signals operate within a narrow concentration window, sufficiently high to activate defense cascades, yet below the threshold of oxidative damage (further detailed below). Constitutive antioxidant overexpression disrupts this window by driving ROS levels below the signaling threshold. If transient, compartment-restricted ROS pulses act as essential upstream triggers of acclimation, then indiscriminate global antioxidant scavenging exerts two severe adverse effects. First, constitutively high antioxidant activity blunts or eliminates stress-induced ROS signal spikes, functionally “desensitizing” plants to environmental stimuli and disabling downstream defense activation cascades. Second, sustained ROS depletion blocks long-distance ROS wave propagation, abolishing systemic preconditioning across the entire plant body. Stress exposure remodels cellular redox status via ROS accumulation, which in turn activates layered defense and acclimation pathways; these redox shifts propagate cell-to-cell via ROS, calcium, and electrical waves to synchronize local and systemic responses [78]. Constitutive over-scavenging disrupts this entire signal cascade.

### 3.4. Eustress, Hormesis, and the Optimal ROS Signaling Window

The relationship between ROS concentration and plant fitness follows a non-linear hormetic dose–response curve, which resolves the apparent contradiction between ROS signaling and ROS toxicity [82]. Basal low-level ROS is required for normal plant growth. Mild, transient increases constitute “eustress”, a beneficial stress that triggers hormetic priming. Only when ROS accumulates excessively and persistently does it overwhelm the cell’s buffering capacity and cause irreversible damage, what might be called “distress” [83]. This distinction matters because the same ROS molecule that triggers beneficial acclimation at low doses becomes toxic at high doses. Thus, under certain conditions, such as acute stress episodes or specific developmental windows, enhanced antioxidant activity is not only beneficial but essential for survival, because it prevents the transition from eustress to distress. Antioxidant systems therefore need to be deployed with care: enough to prevent distress, but not so aggressively that they erase the eustress signals. This balance is context-dependent, varying with stress intensity, tissue type, and developmental stage. Low-dose H_2_O_2_ pretreatment is a classic example of ROS-mediated hormesis; it confers broad cross-tolerance to multiple abiotic stresses [82]. Similarly, low UV-B doses trigger eustress signaling that pre-activates antioxidant defense networks without causing photodamage [84].

**Figure 3:**
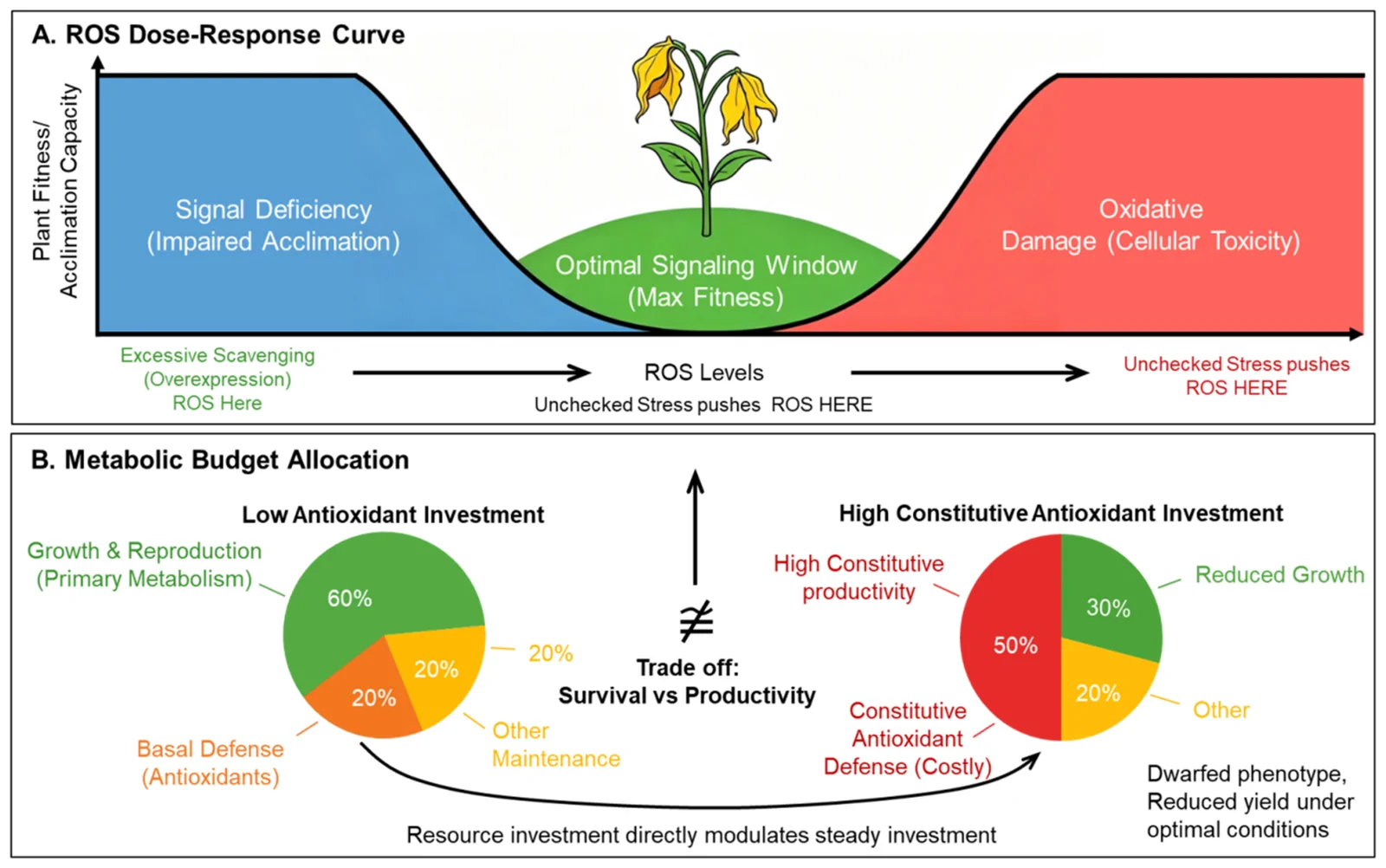
The growth–defense resource trade-off shaped by differential antioxidant investment, combining a hormetic dose–response curve and paired resource allocation pie charts. (**A**) Non-linear hormetic relationship between cellular ROS levels and overall plant fitness/acclimation capacity. The curve forms an inverted U shape divided into three colored zones: blue low-ROS zone representing signal deficiency caused by over-scavenging, green central optimal signaling window marked with maximum plant fitness, and red high-ROS zone associated with oxidative damage and toxicity. Two directional arrows along the *x*-axis indicate that constitutive excessive antioxidant scavenging shifts ROS pools leftward into the signal-deficient range, while unmitigated stress drives ROS accumulation rightward toward toxic oxidative damage. Healthy and wilted plant icons visually represent fitness outcomes in the optimal and toxic ROS ranges, respectively. (**B**) Paired pie charts contrast metabolic resource partitioning under low basal antioxidant investment versus high constitutive antioxidant investment. Under low antioxidant costs, 60% of metabolic resources are allocated to growth and reproductive primary metabolism, alongside 20% basal antioxidant defense and 20% general cellular maintenance. High constitutive antioxidant activity consumes 50% of the metabolic budget for costly persistent antioxidant systems, cutting resources for growth down to only 30% and resulting in dwarfed plant morphology and yield loss under non-stress conditions. An inequality symbol between the two pie charts highlights the core survival-versus-productivity resource trade-off. A curved connecting arrow links panel B to the ROS *x*-axis of panel A to illustrate that metabolic investment in antioxidant machinery directly modulates steady-state cellular ROS concentrations. This figure is a conceptual synthesis of the literature, highlighting the resource trade-off between growth and antioxidant defense.

This biphasic response defines a narrow optimal window for ROS signaling. ROS concentrations must remain sufficiently high to trigger acclimation, but low enough to avoid the oxidation of lipids, proteins, and nucleic acids [85,86]. Antioxidant systems therefore work not as simple ROS erasers but as dynamic rheostats that keep ROS levels within this window [3]. The antioxidant defense system does more than scavenge ROS. It also regulates ROS titers for signaling purposes. Too little antioxidant activity lets ROS climb into toxic territory. Too much constitutive scavenging pushes ROS below the threshold required for signal transduction. The shift we are proposing means giving up on maximizing ROS removal and instead maintaining a dynamically balanced redox state (Figure 3), achieved through the balance between ROS production and scavenging [87,88].

## 4. The Metabolic Cost of Constitutive Antioxidant Defense

Beyond the direct interference with signal transduction, constitutive antioxidant overexpression creates a second problem: resource allocation. This metabolic burden compounds the signaling defects we discussed above. When plants perceive pathogens or stress, they mount a range of defensive responses, including mitogen-activated protein kinase (MAPK) activation, ROS burst, transcriptional reprogramming, and the synthesis of defense-related compounds. These are not passive consequences of stress perception; they are active and organized cellular events that require substantial metabolic investment. Mounting these responses to environmental changes is energetically demanding and metabolically costly [89]. Antioxidant systems are no exception. Synthesizing and maintaining enzymes like SOD, CAT, and APX require carbon skeletons, reducing power (NADPH), and ATP. Non-enzymatic antioxidants like ascorbate, glutathione, and phenolic compounds are even more expensive. A large fraction of photosynthetically fixed carbon flows into the phenylpropanoid pathway, producing phenolic compounds, flavonoids, and lignin precursors that contribute to both structural defense and antioxidant buffering. This represents a major diversion of carbon from primary metabolism to secondary defense pathways [90]. Plant defensive traits are costly for plants due to the energy drain from growth toward defensive metabolite production.

When resources are limited, as they are in most natural and agricultural settings, these metabolic costs can become a major constraint. Carbon, nitrogen, and energy allocated to antioxidant defense cannot simultaneously be allocated to growth, reproduction, or other fitness-related functions. This generates a canonical growth–defense resource trade-off: investment in protection comes at the expense of productivity [91,92]. Notably, plants engineered to exhibit increased defense responses tend to suffer from reduced growth [93]. This is often not a failure of engineering itself; it reflects a fundamental biological constraint. The plant has a finite resource budget, and defense consumes resources that would otherwise fuel growth. In the context of antioxidant defense, this resource trade-off is particularly acute because antioxidant systems must be maintained continuously and they cannot be turned on and off instantaneously in response to stress.

Recent advances have begun to elucidate the molecular mechanisms underlying these resource allocation decisions. Within the framework of the growth–defense resource trade-off, target of rapamycin (TOR) and SNF-related kinase 1 (SnRK1) have emerged as two central kinases that connect metabolic status to cellular outputs [89]. TOR is activated under nutrient-sufficient conditions and promotes anabolic growth processes, whereas SnRK1 becomes activated during energy deprivation and redirects metabolism toward catabolic pathways such as autophagy and nutrient recycling [94]. TOR acts as a molecular switch for the activation of cell proliferation and plant growth at the expense of cellular immunity [95]. However, defense activation is markedly restricted under sugar-starvation conditions where SnRK1 is activated [89], indicating that plants actively suppress defense signaling when cellular energy availability is insufficient. Direct molecular links between these energy sensors and antioxidant gene expression have emerged: SnRK1 activation under carbon starvation suppresses the expression of several antioxidant enzyme genes, including cytosolic APX and Cu/Zn-SOD, while TOR signaling promotes the translation of ROS-scavenging enzymes under favorable growth conditions [94]. This regulatory system appears to ensure that antioxidant investment is scaled to cellular energy availability, potentially preventing unnecessary defense expenditure when resources are limited.

This molecular regulatory network underscores that the growth–defense resource trade-off is not merely a conceptual framework but is actively orchestrated at the signaling level. The consequences of this resource trade-off are evident at multiple scales. At the biochemical level, the activation of phenylpropanoid pathways for phenolic antioxidant synthesis diverts carbon from primary metabolism [90]. At the physiological level, constitutive antioxidant investment can reduce photosynthetic capacity and biomass accumulation [96]. At the evolutionary level, the resource trade-off may help explain why plants have not evolved to maximize antioxidant capacity, as the fitness costs of constitutive defense outweigh the benefits in the absence of continuous stress. Plants have evolved strategies to minimize the metabolic burden of constitutive defense, including prioritizing first-line defenses in the apoplastic space involving ascorbate, defensins, and small peptides, before cellular processes are affected [97]. This prioritization strategy reflects an evolutionary optimization—investing just enough in defense to ensure survival, but not so much that growth and reproduction are fatally compromised.

This growth–defense resource trade-off has direct implications for crop improvement. Strategies that aim to enhance stress tolerance by constitutively elevating antioxidant systems may succeed in the laboratory under controlled conditions, but often fail in the field where plants must balance survival with productivity. A plant that survives drought by investing heavily in antioxidant defense but produces little grain has not solved the farmer’s problem. The challenge is not to maximize survival at any cost, but to optimize the survival–productivity balance under stress. Plants employ several strategies to reduce the high metabolic costs associated with chemical defense [98], suggesting that future engineering efforts should focus not on maximizing antioxidant capacity, but on optimizing the timing, location, and magnitude of antioxidant deployment to minimize the associated growth penalty while maintaining adequate stress protection.

**Figure 4:**
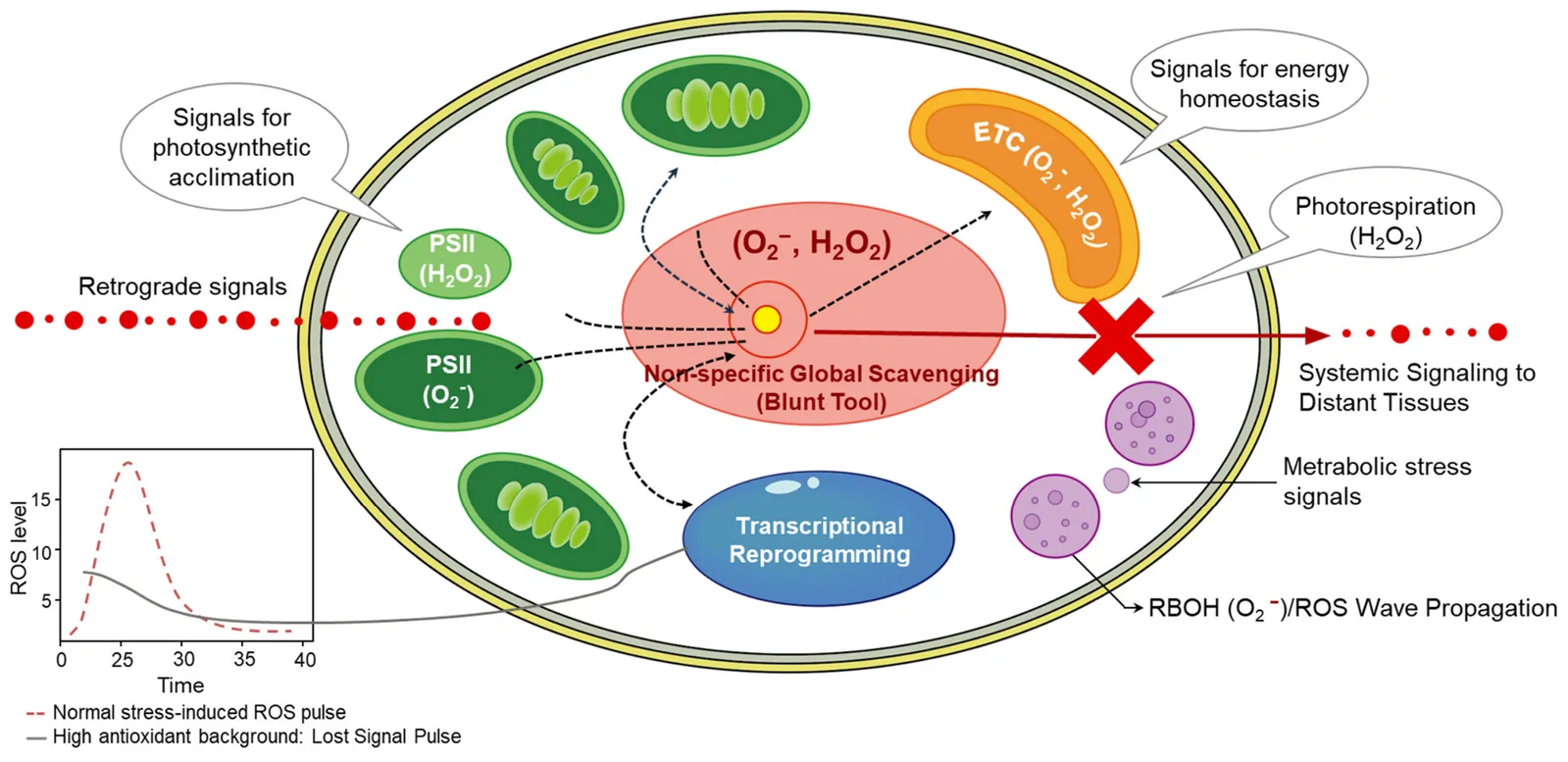
Subcellular compartmentalization and temporal dynamics of ROS signaling reveal the drawbacks of non-specific antioxidant scavenging. The main schematic illustrates distinct subcellular compartments, including the chloroplast, mitochondrion, peroxisome, and apoplast, that generate unique ROS species with specific signaling functions. Chloroplasts produce singlet oxygen (^1^O_2_) at PSII and H_2_O_2_ at PSI to mediate photosynthetic acclimation; mitochondria generate superoxide (O_2_•^−^) and H_2_O_2_ via the electron transport chain to regulate energy homeostasis; peroxisomal ROS supports photorespiration-associated signaling; and apoplastic ROS produced by RBOHs drives cell-autonomous responses and systemic ROS wave propagation. Spatially specific retrograde signals from these organelles converge on the nucleus to orchestrate stress-induced transcriptional reprogramming. Non-specific, global antioxidant scavenging (red shaded cloud) acts as a blunt tool that indiscriminately eliminates compartment-specific ROS signals, abolishing tailored organellar retrograde signaling and blocking systemic ROS wave transmission. The temporal inset (bottom right) demonstrates that under normal stress perception, stress triggers a transient ROS pulse that propagates as a signaling wave, activating downstream acclimation responses. Under constitutive high antioxidant activity, the same stress input generates a blunted ROS signal with reduced peak amplitude and prolonged duration, failing to reach the threshold required for systemic acquired acclimation. The flattened waveform illustrates how constitutive scavenging erases both the spatial and temporal information encoded in ROS signals. Spatiotemporally resolved monitoring, using roGFP2-based fluorescent probes and real-time imaging of H_2_O_2_ dynamics, enables in vivo tracking of these ROS pulses within specific subcellular compartments, as described later in this review. This figure presents a conceptual model integrating spatial and temporal dimensions of ROS signaling as described in the literature.

The hormetic relationship depicted in Figure 4, an inverted U-shaped curve, formally resolves the apparent duality of ROS: basal ROS is required for growth (left slope), moderate transient elevation constitutes “eustress” that triggers acclimation (peak zone), and only excessive, persistent accumulation causes irreversible damage (right slope). The paired pie charts in Figure 4 quantify the metabolic reality underlying this resource trade-off: high constitutive antioxidant investment consumes up to 50% of the metabolic budget, severely constraining resources available for growth and reproduction. These quantitative insights establish that antioxidant optimization is fundamentally a resource allocation problem, not merely a biochemical one. Having established the metabolic constraints on constitutive antioxidant defense, we next examine the equally critical dimensions of spatial and temporal specificity that govern ROS signaling functions.

## 5. When and Where: The Spatial and Temporal Specificity of ROS Signaling

The third core argument concerns spatial and temporal specificity. This is a dimension that constitutively high antioxidant activity inevitably disrupts. ROS are not evenly distributed across plant cells and tissues; they are generated in specific subcellular compartments, including chloroplasts, mitochondria, peroxisomes, the apoplast, and the plasma membrane (Figure 4).

Each compartment produces distinct ROS species through different biochemical reactions, and each discrete ROS pool executes out compartment-specific signaling functions [2,99]. The spatial distribution, temporal fluctuation, and chemical identity of ROS collectively encode information about the type and intensity of stress, allowing cells to mount targeted responses [100]. Chloroplasts, mitochondria, and peroxisomes are the main ROS hotspots, driven by photosynthesis, photorespiration, and respiration, respectively. Fine control over organelle-resident ROS pools directly affects the efficiency of downstream stress acclimation [101].

### 5.1. Compartment-Specific ROS Production and Retrograde Signaling

Individual organelles generate characteristic ROS signatures that initiate dedicated plastid/mitochondrion-to-nucleus retrograde signaling cascades. In chloroplasts, photosystem II generates singlet oxygen (^1^O_2_), while photosystem I releases H_2_O_2_ [2]. Chloroplast-derived ROS serve dual functions: they locally modulate photosynthetic metabolism and act as retrograde messengers to reshape nuclear transcriptomes [2]. In *Arabidopsis thaliana*, the EXECUTER (EX1/EX2) protein family represents the central transduction module for 1O2-triggered retrograde signals [102]. This signaling axis relies on EX1/EX2 and nuclear WRKY transcription factors, with ABI4 acting as a key downstream repressor/activator balancing acclimation and programmed cell death [103]. Sub-lethal concentrations of ^1^O_2_ initiate chloroplast retrograde signaling to activate protective acclimation pathways; excessive singlet oxygen accumulation instead drives cell death [104,105].

Mitochondrial ROS originate from electron transport chain leakage and coordinate respiratory function, hormone signaling, retrograde communication, immunity, and programmed cell death [106]. Peroxisomal ROS are produced during photorespiration and fatty acid catabolism; ROS/RNS dynamics within peroxisomes remodel organelle plasticity and mediate systemic plant stress responses [107]. Across chloroplasts, mitochondria, and peroxisomes, cellular machinery maintains a narrow ROS concentration window to preserve signaling activity while avoiding irreversible oxidative damage [4]. Chloroplast retrograde signals further stimulate the biosynthesis of defensive secondary metabolites and stress hormones to facilitate whole-plant environmental acclimation [108].

This compartmental separation creates a fundamental functional dichotomy: identical ROS molecules can act as essential signaling cues in one subcellular location yet toxic oxidants in another, determined by local redox buffering capacity, target protein abundance, and co-signaling partners. Cysteine thiol molecular switches function as compartment-resolved ROS sensors, converting oxidative modifications into conformational changes that regulate protein activity, subcellular localization, and downstream nuclear transcription [2]. Critically, constitutive global antioxidant overexpression can act as an indiscriminate buffer that erases all compartmental ROS gradients simultaneously. It cannot distinguish between signal-mediating ROS and cytotoxic ROS, eliminating both pools and abolishing compartment-specific retrograde communication entirely.

Complementing spatial compartmentalization, ROS signaling also exhibits rigid temporal dynamics that are equally critical. Stress-triggered ROS pulses propagate as well as cell-to-cell waves to coordinate systemic acquired accumulation, as described earlier. Constitutive antioxidant overexpression dampens these transient spikes and slows wave propagation, thereby disrupting both the “where” and the “when” of ROS signals.

### 5.2. Real-Time Imaging: Uncovering Hidden Spatiotemporal ROS Dynamics

Traditional endpoint measurements of antioxidant enzyme activity only capture a static average of redox status, missing the fine-grained subcellular and temporal fluctuations of ROS signals. Live-cell real-time imaging has overcome this limitation, making it possible to visualize ROS generation, scavenging, and signaling in intact plant tissues at the subcellular level [3].

Redox-sensitive GFP (roGFP)-based genetically encoded biosensors enable the quantitative tracking of H_2_O_2_ fluctuations in vivo [109]. The optimized roGFP2-PRXIIB probe, fused with endogenous type II peroxiredoxin, exhibits superior kinetic responsiveness and signal stability, allowing for robust visualization of H_2_O_2_ dynamics under both abiotic and biotic stress [109]. Targeting this probe to separate subcellular compartments (cytosol, nucleus, mitochondria, chloroplasts) revealed compartment-unique temporal H_2_O_2_ burst patterns during pattern-triggered and effector-triggered immunity, confirming spatially segregated signal programs [109].

Collectively, in vivo imaging approaches demonstrate that ROS signals encode nuanced information about stress type, intensity, and duration via tightly controlled spatiotemporal patterning, rather than operating as simple binary on/off switches [3]. These imaging advances reinforce that balanced ROS production and scavenging across compartments is a prerequisite for optimal plant fitness [4].

Taken together, the spatial compartmentalization and temporal pulsing of ROS signals redefine the core limitation of blanket antioxidant overexpression. Plant redox regulation cannot be simplified to a single universal antioxidant dosage metric; instead, two critical dimensions govern functional outcomes: the location (which compartment generates which ROS species) and the timing (whether ROS signals arrive as transient pulses or sustained elevations). Tissue- or organelle-targeted, stress-inducible antioxidant enhancement can effectively mitigate local oxidative damage without disrupting global signaling. In contrast, constitutive, cell-wide over-scavenging erases compartmental retrograde signals and blunts dynamic ROS wave propagation, undermining plant acclimation capacity. This paradigm necessitates a shift toward precision redox engineering that tailors antioxidant activity to specific subcellular locations and stress time windows, rather than pursuing uniform, maximal ROS scavenging across the entire plant.

As illustrated in Figure 4, each subcellular compartment generates unique ROS species with specific signaling outputs, and non-specific global scavenging (red shaded cloud) disrupts both organellar retrograde communication and systemic ROS wave propagation. Having established the spatiotemporal constraints on antioxidant deployment, we next examine in Section 6 the boundary conditions under which antioxidant systems remain indispensable, before outlining our precision redox framework.

## 6. Boundary Conditions: When Are Antioxidants Beneficial?

We have laid out three mechanisms by which constitutive antioxidant activity impairs stress acclimation. However, it is equally important to recognize that antioxidants are not inherently bad. Whether they help or hurt depends on context. To keep the argument from becoming one-sided, here we describe specific physiological situations where elevated antioxidant capacity is clearly beneficial, where it crosses the line from harmful over-scavenging to necessary redox buffering. The same antioxidant systems that impair acclimation when constitutively overexpressed are indispensable for survival under certain conditions.

Building on the conceptual “optimal ROS signaling window” introduced in the preceding section, recent quantitative in vivo imaging studies have begun to define its numerical boundaries. What constitutes “excessive” ROS accumulation that exceeds the signaling window? While the precise threshold varies by species, tissue, and developmental stage, in vivo imaging studies have provided quantitative reference points. In Arabidopsis thaliana, basal cytosolic H_2_O_2_ concentrations are maintained at approximately 0.1–0.5 µM under optimal growth conditions [110]. Mild stress-induced elevations to 1–5 µM trigger hormetic priming and acclimation responses without causing significant oxidative damage [111]. However, when H_2_O_2_ concentrations exceed 10–20 µM persistently, lipid peroxidation markers (MDA) and protein carbonylation increase sharply, culminating in cell death [112]. These quantitative boundaries define the “permissive signaling window”: ROS levels must remain above the signal threshold (to trigger acclimation) yet below the toxicity threshold (to avoid irreversible damage). Constitutive antioxidant overexpression that drives ROS below the signal threshold is as detrimental as insufficient scavenging that allows ROS to accumulate beyond the toxicity threshold.

With this quantitative framework in mind, we can now delineate the specific physiological scenarios where elevated antioxidant capacity confers clear fitness advantages. Antioxidants become irreplaceable under acute, extreme abiotic stress that pushes cellular ROS concentrations far beyond the permissible signaling window [64]. Balanced ROS generation and clearance support unimpaired cellular development; however, severe stress triggers massive ROS bursts that overwhelm native buffering capacity, triggering irreversible damage to lipids, proteins, and nucleic acids once ROS exceeds toxic thresholds [64]. Without sufficient enzymatic and non-enzymatic antioxidant pools to neutralize excess ROS, oxidative injury accumulates progressively and culminates in cell death [11]. Under harsh climatic or edaphic stress, coordinated antioxidant activation restores redox equilibrium and alleviates lethal oxidative lesions. In such toxic ROS regimes, enhanced antioxidant activity represents a vital survival mechanism rather than a disadvantage. Beyond acute stress scenarios, antioxidants are also indispensable during specific developmental stages. Maturing seeds, senescing organs, and meristematic tissues generate intrinsically high basal ROS loads that require robust antioxidant buffering to sustain normal developmental progression [113,114,115]. For example, the ascorbate–glutathione cycle maintains redox balance during seed maturation and germination [114], while ascorbate functions as a multifunctional regulator of cell division and differentiation throughout the plant life cycle [115]. These cases illustrate that antioxidant systems are not merely emergency stress responders but core components of plant development, further emphasizing that the question is not whether antioxidants are good or bad, but when, where, and to what extent they are deployed.

A clear functional distinction separates inducible, compartment-targeted upregulation and permanent, cell-wide constitutive overexpression as two modes of antioxidant regulation. Stress-triggered antioxidant induction is a refined tuning mechanism that adjusts ROS levels locally within specific subcellular domains and temporary timeframes. Constitutive expression, in contrast, is a blunt tool that wipes out ROS signals and wastes resources. The functional distinction between inducible and constitutive antioxidant expression is not merely qualitative but can be quantified. Stress-inducible promoters such as SWPA2 drive antioxidant gene expression up to 10- to 50-fold upon stress perception, with expression returning to basal levels within hours of stress relief [116]. In contrast, constitutive overexpression driven by the CaMV 35S promoter maintains 5- to 20-fold elevated antioxidant activity continuously, regardless of stress status [117]. This sustained elevation incurs a measurable growth penalty: transgenic lines with constitutive antioxidant overexpression typically show 15–30% reduction in biomass under non-stress conditions compared to the wild-type, whereas inducible expression systems show no significant growth reduction while providing comparable stress protection (Figure 4) [116,117]. Adaptive stress acclimation relies on this targeted induction logic [27]. During acclimation priming, plants selectively upregulate subsets of antioxidant enzymes in discrete organelles rather than globally boosting all antioxidant pathways. For example, ABA–ROS–antioxidant regulatory modules mitigate osmotic injury in acclimated seedlings [118]; cold acclimation induces coordinated antioxidant elevation alongside improved photosynthetic efficiency and hormone homeostasis to counter chilling damage [119]. This contrast highlights that antioxidant efficacy hinges on timing and spatial specificity, not total activity magnitude. Across plant defense research, a consistent distinction exists: constitutive baseline defense incurs persistent metabolic costs, while stress-inducible defense delivers protection without sustained growth penalties.

Specialized developmental stages and tissue types constitute a second category where high antioxidant reserves are mandatory. Maturing seeds, senescent organs and meristematic tissues intrinsically generate high basal ROS loads, requiring robust antioxidant buffering to sustain developmental progression. ROS govern critical seed germination processes including cell wall loosening, endosperm breakdown and ABA signal suppression, yet unchecked ROS accumulation compromises seed viability [113]. Comprehensive antioxidant networks, including detoxification enzymes and low-molecular redox metabolites, preserve seed longevity during dormancy [114]. Ascorbate (vitamin C) acts as a multifunctional redox regulator indispensable for photosynthesis, cell division and organ differentiation throughout the plant life cycle [115]. These developmental functions establish that antioxidants are not merely emergency stress response machinery, but core components governing all phases of plant growth and reproduction.

Enzymatic and small-molecule non-enzymatic antioxidants fulfill divergent, non-overlapping physiological roles. Low-molecular redox metabolites, including ascorbate and glutathione in particular, operate simultaneously as ROS scavengers and central nodes of redox signal transduction [115]. The ascorbate–glutathione cycle forms the backbone of plant redox networks, linking central energy metabolism to downstream signaling cascades with multifunctional secondary activities beyond ROS detoxification. Cycle enzymes undergo reversible oxidative post-translational modifications and interact with a broad spectrum of signaling proteins, possessing well-documented moonlighting regulatory functions [115]. Collectively, ascorbate and glutathione do not merely limit ROS signal duration; they mediate widespread redox-dependent transcriptional and post-translational reprogramming across cellular compartments, far exceeding simple oxidative neutralization.

To guide experimental design and breeding strategies, we propose a simple decision framework for evaluating when antioxidant enhancement is beneficial versus counterproductive. Specifically, when stress is acute and severe such that ROS concentrations far exceed the toxicity threshold, enhanced antioxidant capacity is beneficial and should involve rapid induction targeted to damaged compartments. In contrast, when stress is chronic or moderate such that ROS levels remain within or slightly above the signaling window, antioxidant enhancement is likely counterproductive; in this scenario, preserving ROS signaling and focusing on signal perception and transduction should be prioritized. When the goal is field productivity under fluctuating stress, inducible and compartment-targeted antioxidant systems are preferable over constitutive overexpression. Finally, when the experimental readout is a single endpoint enzyme activity measurement, the results should be interpreted with caution and complemented with dynamic imaging and phenotypic assays.

In practice, antioxidant systems are neither universally detrimental nor universally beneficial; their fitness outcome is strictly context-dependent, determined by stress intensity, developmental stage, subcellular localization, and temporal expression pattern. Our analysis does not argue for eliminating antioxidant research or for dismissing the protective functions of antioxidants. Rather, we emphasize that antioxidants are essential under specific conditions, and the critical challenge is to distinguish these beneficial contexts from those in which constitutive overexpression becomes counterproductive. As our analysis has demonstrated, the apparent paradox of ROS dual functionality, specifically their roles as both signals and toxins, is resolved by recognizing that context determines outcome.

## 7. Toward a New Framework: From Scavenging to Signaling 

Drawing together the mechanistic paradoxes, signal interference, metabolic trade-offs, and spatiotemporal constraints discussed above, this review argues for a shift in how we think about plant antioxidant research. Instead of treating antioxidant enzymes and metabolites as bulk ROS scavengers to be maximized, we should see them as components of a dynamic redox network that keeps ROS concentrations within a narrow physiological window [120]. Advances in computational modeling, multi-omics, and plant biotechnology are pushing the field into a new phase, one that leaves behind the old “more antioxidants equal tolerance” logic [13]. This conceptual shift has implications for both basic redox research and applied crop breeding. We lay out five interconnected directions below.

### 7.1. Prioritize Redox Responsiveness over Constitutive High Antioxidant Abundance

The revised engineering goal is to optimize the dynamic responsiveness of the redox network, not to permanently raise the baseline antioxidant levels. Genotypes with moderate constitutive antioxidant levels but strong, rapid stress-inducible upregulation perform better than lines with static high basal antioxidant capacity because the latter blunts essential ROS signal pulses [13]. A well-coordinated balance between antioxidant buffering, nutrient homeostasis, and ROS signal supports whole-plant resilience under fluctuating abiotic stress [121]. The integrated redox network dual-functionally mitigates irreversible oxidative damage and preserves transient ROS signals required for acclimation priming, as illustrated in the conceptual redox window model (Figure 1). Accordingly, crop improvement research must reframe its central question from how to boost static antioxidant content to how to engineer plants with finely tuned, stress-responsive redox circuitry.

### 7.2. Shift from Single-Gene Manipulation to Coordinated Network Tuning

Traditional single-transgene overexpression strategies often fail to improve field stress tolerance because they disrupt the balanced connectivity of the interconnected antioxidant regulatory network. The full antioxidant machinery consists of coordinated enzymatic modules (SOD, CAT, APX etc.) and non-enzymatic low-molecular redox buffers (ascorbate, glutathione, flavonoids), whose balanced stoichiometry is essential for stable redox homeostasis [13]. Future genetic interventions should target coordinated network optimization rather than isolated individual genes. Systems biology tools integrating transcriptomic, proteomic, and metabolomic profiling enable a comprehensive dissection of antioxidant pathway dynamics across crop genotypes and stress histories [122]. Multi-omics datasets uncover layered ROS–Ca^2+^–phytohormone signal axes that orchestrate systemic plant acclimation [121]. Further fusion of redox proteomics with artificial intelligence predictive platforms enables systematic identification of core network nodes, accelerating the development of precision agriculture varieties resilient to climate fluctuation [123].

### 7.3. Oxidative Post-Translational Modifications (oxiPTMs): Molecular-Level Reversible Redox Switches

Oxidative post-translational modifications (oxiPTMs) represent the most refined native mechanism for spatiotemporally precise antioxidant regulation. They offer actionable molecular targets for crop engineering under climate stress [120]. ROS, RNS, and RSS modulate antioxidant protein conformation, subcellular localization, and catalytic activity via cysteine residue-targeted modifications. These modifications form the core molecular infrastructure of environmental signal perception [120]. Recent comprehensive reviews have systematically cataloged plant oxiPTMs targeting antioxidant and redox proteins, detailing the specific conditions under which these modifications occur and their effects on catalytic activities, protein structure, and subcellular localization [120]. The reversible nature of most thiol-based oxiPTMs is key to their function in plant stress responses [120].

ROS-induced Oxi-PTMs, particularly S-glutathionylation, are increasingly recognized as molecular switches that activate or inhibit key signaling proteins and transcription factors [124]. Major thiol-based oxiPTMs include S-sulfenylation, S-glutathionylation, S-nitrosation, persulfidation, S-cyanylation and S-acylation [125]. Unlike slow transcriptional upregulation, these modifications act rapidly and reversibly to fine-tune antioxidant activity in discrete subcellular compartments without globally erasing ROS signals. Accumulating evidence confirms that H_2_O_2_ executes nearly all of its signaling functions through cysteine-targeted oxiPTMs, which can be treated as universal molecular switches governing plant stress responses [124]. Native oxiPTM machinery solves the core flaw of constitutive antioxidant overexpression by enabling transient, localized ROS buffering while preserving long-distance signal propagation. Complementing these protein-level switches, real-time imaging tools provide the spatiotemporal resolution needed to observe their functional consequences in vivo.

### 7.4. Subcellular Real-Time ROS Imaging: Visualizing Hidden Spatiotemporal Signal Dynamics

Real-time live-cell imaging technologies eliminate the limitations of traditional endpoint antioxidant enzyme measurements, which only capture static average redox status and obscure compartment-specific transient ROS pulses [3]. Redox-sensitive GFP (roGFP) genetically encoded biosensors support non-invasive quantitative tracking of intracellular thiol redox balance and H_2_O_2_ fluctuations [126]. The optimized roGFP2-PRXIIB fusion probe exhibits superior kinetic sensitivity and signal stability, enabling the clear visualization of H_2_O_2_ bursts triggered by both abiotic and biotic stress [109]. The roGFP2-PRXIIB probe, when targeted to cytosol, nuclei, mitochondria, and chloroplasts, has revealed distinct temporal patterns of H_2_O_2_ accumulation during pattern-triggered and effector-triggered immune responses across different subcellular compartments, directly confirming the spatially segregated nature of ROS signaling programs [109]. Combined with genetic and pharmacological perturbation assays, these in vivo imaging tools allow researchers to dissect retrograde organelle signaling and rationally design spatially restricted antioxidant modification strategies [3,127]. While imaging reveals the natural dynamics of ROS signals, synthetic biology offers the means to rationally reprogram these dynamics.

To assist readers in navigating the methodological landscape, we summarize the key techniques for studying plant redox regulation in Table 2, highlighting their applications, benefits, and current limitations.

### 7.5. Synthetic Biology and Multi-Omics Integration for Programmable Antioxidant Responses

Synthetic biology delivers modular genetic tools to achieve spatiotemporally programmable antioxidant expression, breaking the limitations of constitutive overexpression constructs [13]. Stress-inducible promoter systems such as oxidative stress-responsive SWPA2 drive antioxidant gene expression exclusively under adverse conditions, drastically cutting persistent metabolic growth penalties while retaining sufficient oxidative damage protection [128].

Several examples show that inducible antioxidant regulation works in crops. In transgenic potato (*Solanum tuberosum*), co-expression of CuZnSOD and APX under the oxidative stress-inducible SWPA2 promoter conferred enhanced tolerance to methyl viologen-induced oxidative stress at the leaf-disc, plantlet, and whole-plant levels, without the growth penalties typically associated with constitutive overexpression [116,128]. In transgenic sweet potato, the same strategy using the SWPA2 promoter driving CuZnSOD and APX expression similarly improved tolerance to multiple environmental stresses [116]. In transgenic rice, overexpression of the dehydroascorbate reductase gene *OsDHAR1* under the control of the stress-inducible SWPA2 promoter improved salt tolerance, enhanced photosynthetic ability and membrane stability, and maintained grain yield under paddy-field conditions [129]. In transgenic poplar, AtNDPK2 expression under SWPA2 improved oxidative stress tolerance and promoted growth [117]. Collectively, these cases demonstrate that inducible regulation can achieve a better balance between stress protection and growth than constitutive overexpression.

Organelle-targeted antioxidant transgenes add another layer, enabling localized ROS detoxification in damage-prone compartments without disrupting cell-wide systemic ROS wave transmission. When paired with multi-omics network mapping, synthetic gene circuits can be customized to tune entire redox pathways rather than single enzymes.

Complementary multi-omics integration provides a systems-level roadmap for synthetic engineering. Combined transcriptome, proteome, and metabolome datasets resolve complex redox regulatory motifs and identify master regulatory genes controlling drought tolerance and yield stability in staple crops such as rice [109]. Cutting-edge computational tools including CysQuant, BiGRUD-SA, DLF-Sul, and Plant PTM Viewer leverage machine learning to predict redox-sensitive cysteine residues and reconstruct complete redox signaling networks from proteomic data [123]. The combination of multi-omics profiling, AI predictive modeling and synthetic biology circuit design establishes a complete workflow for precision redox engineering in crops. This workflow consists of three phrases: design, which employs synthetic gene circuits for spatiotemporal control; predict, which uses AI modeling of redox network behavior; and validate, which applies multi-omics profiling to engineered lines.

**Figure 5:**
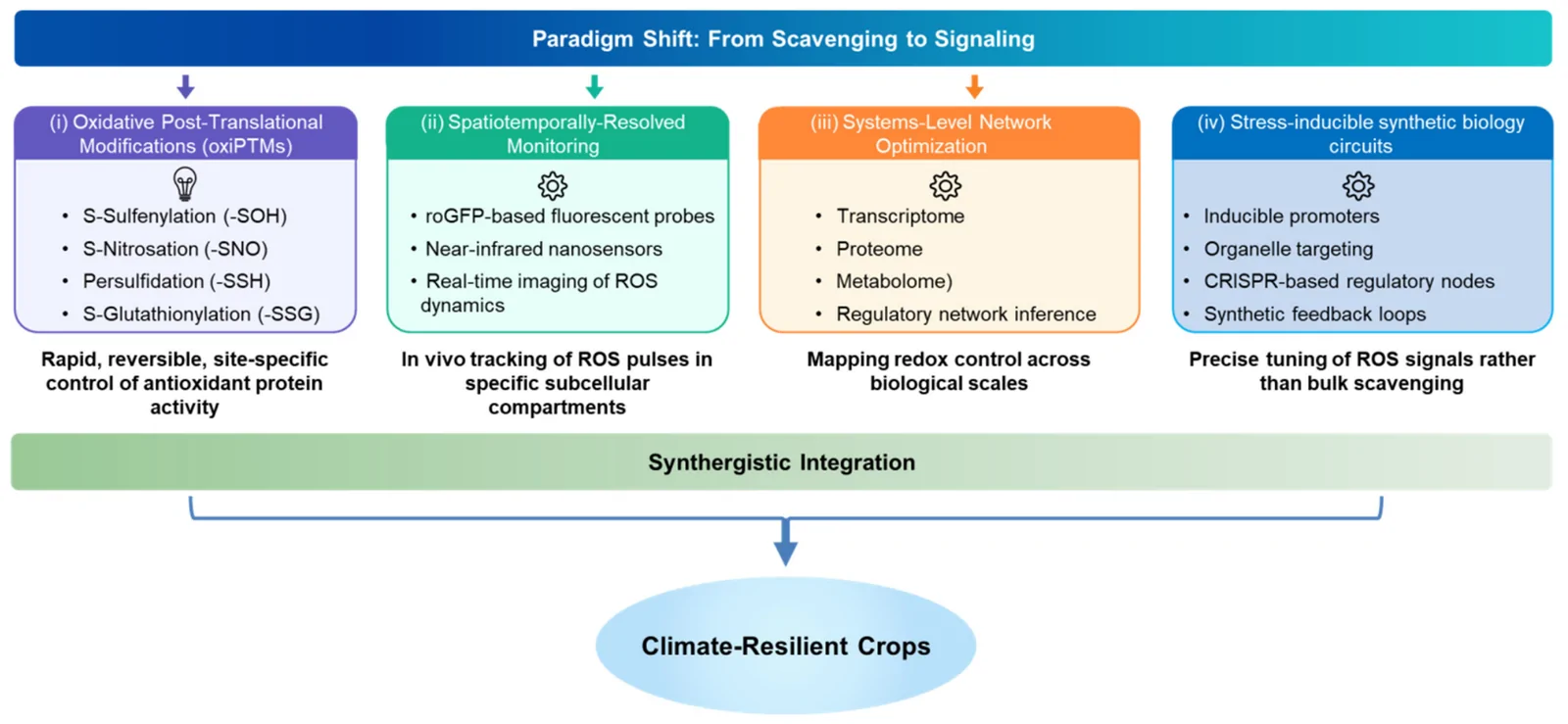
A proposed framework for advancing plant redox biology from descriptive antioxidant measurements to precision redox regulation. Four complementary pillars are envisioned to support this transition. (i) Oxidative post-translational modifications (oxiPTMs), including S-sulfenylation, S-nitrosation, persulfidation, and S-glutathionylation, provide a mechanism for rapid, reversible, and site-specific control of antioxidant protein activity. (ii) Spatiotemporally resolved monitoring, using roGFP2-based fluorescent probes and real-time imaging of H_2_O_2_ and O_2_•^−^ dynamics enables the in vivo tracking of ROS pulses within specific subcellular compartments. (iii) Network-wide multi-omics analysis, integrating transcriptomic, proteomic, and metabolomic profiling with AI predictive platforms, enables the systematic identification of core regulatory nodes. (iv) Stress-inducible synthetic biology circuits, including inducible promoters and organelle-targeting transgenes, enable spatiotemporally programmable antioxidant expression while minimizing growth penalties. The synergistic integration of these four pillars offers a path toward developing climate-resilient crops that balance stress survival with productivity under field conditions. This framework is a conceptual synthesis of the precision redox regulation strategies discussed in the literature.

Collectively, as illustrated in Figure 5, this new paradigm shifts the central goal from blanket ROS scavenging to multi-dimensional precision redox regulation, requiring the coordinated deployment of four core research pillars: reversible oxiPTM molecular switches for rapid and reversible protein-level control (i); subcellular real-time ROS imaging for visualizing spatiotemporal signal dynamics (ii); network-wide multi-omics analysis integrated with AI predictive platforms for the systematic identification of core regulatory nodes (iii); and stress-inducible synthetic biology circuits for spatiotemporally programmable antioxidant expression with minimal growth penalties (iv). The synergistic integration of these four pillars, shown converging in the center of the figure, enables a rational manipulation of antioxidant activity at precise locations and time points, striking a sustainable balance between plant survival under environmental stress and crop productivity under field conditions. This precision redox framework lays a solid theoretical and technical foundation for breeding climate-resilient crops suited to future variable climates, and we elaborate on its translational implications in the following section.

## 8. Conclusions and Implications

### 8.1. Core Conclusions

The main conclusion is straightforward but challenges a long-held assumption: more antioxidants do not always mean better stress acclimation. The common practice of using antioxidant enzyme activity as a biomarker for stress tolerance is not just an oversimplification—it is often misleading, as in vivo and transgenic evidence frequently contradicts it [12]. Consistent with Xu et al. [12], constitutively elevated antioxidant machinery disrupts stress-triggered ROS signaling and weakens plant adaptive capacity [99]. Overproducing antioxidants permanently and across the whole plant leads to three interconnected problems [10]. These include the suppression of essential stress signals, diversion of metabolic resource away from growth and reproduction, and breakdown of the spatiotemporal ROS gradients needed for SAA. None of this means that antioxidant systems are useless. As discussed earlier, they are essential for survival under acute, severe stress, when ROS levels exceed the signaling threshold and cause oxidative damage. What we are challenging is the untested assumption that more antioxidants always mean better fitness, and the breeding strategies built on that assumption. ROS are inherently two-faced in plant physiology. They drive adaptive signaling at moderate levels and cause damage at high levels. Plants manage this through tightly coordinated, multi-layered redox networks that balance these opposing effects [13,121]. The goal should be to maintain a delicate, context-dependent redox balance, not to eliminate ROS through unrestrained antioxidant overexpression.

### 8.2. Current Experimental Constraints and Practical Challenges

Several practical challenges limit the translation of these ideas from the lab to the field. (i) What works in controlled environments often does not translate to field conditions. In the field, plants face multiple interacting stresses at once, such as drought plus heat, or salinity plus pathogens, and the performance of antioxidant-enhanced lines observed in growth chambers frequently does not hold up in the field. (ii) Achieving precise spatiotemporal control of antioxidant expression in intact plants is technically difficult. Even with stress-inducible promoters like SWPA2, the timing and magnitude of induction depend on stress severity, tissue type, and developmental stage, making consistent control hard to achieve. (iii) The field lacks standardized protocols for measuring ROS dynamics in real-time. Different labs use different probes, imaging setups, and analysis pipelines, which makes it hard to compare results across studies. (iv) The resource trade-off between stress tolerance and yield is fundamental. Even with inducible systems, any investment in defense draws resources away from primary metabolism. There are limits to how much stress tolerance can be improved without sacrificing productivity. These challenges do not make precision redox engineering less promising. They simply mean that we need continued methodological development, better standardization, and more field-based phenotyping to bridge the gap between laboratory insights and practical crop improvement.

### 8.3. Implications for Research Practice and Translational Crop Breeding

The findings have concrete implications for both research practice and crop breeding.

In research practice, the routine use of static endpoint measurements of antioxidant enzyme activity as a primary readout of stress tolerance needs rethinking. Single time-point measurements capture only a snapshot of a continuously changing system; they need to be complemented with dynamic assays that capture temporal and spatial ROS fluctuations. Transgenic strategies that aim for constitutive overexpression should be reconsidered in favor of stress-inducible, tissue-specific, or organelle-targeted approaches. The growing toolkit of roGFP-based sensors, oxiPTM profiling [35], and multi-omics network analysis offers new ways to understand what determines antioxidant efficacy. Adopting these methods would help move the field from the “more-is-better” mindset toward a precision redox framework. In crop breeding, constitutive antioxidant overexpression often fails to deliver consistent stress tolerance and yield under field conditions, where plants have to balance survival and productivity at the same time. Growth and defense compete for limited carbon, nitrogen, and energy, creating a trade-off that affects vegetative development, reproductive output, and overall fitness [10,130,131].

Modern crop improvement needs more refined approaches: dynamically tuning redox homeostasis, restricting antioxidant activation to specific compartments and stress windows, and accounting for resource trade-offs in genetic design. Concrete examples like the SWPA2-driven expression of CuZnSOD and APX in potato and sweet potato, and OsDHAR1 overexpression in rice under stress-inducible control, show that this approach is feasible. The tools to implement this precision framework are already available, including multi-omics for mapping redox regulatory hubs [121,132], CRISPR-Cas for targeted gene editing [133], and AI combined with systems modeling for predicting antioxidant network behavior under fluctuating conditions. By bringing together these methodological and conceptual advances, the plant redox community can move from static, single-gene overexpression to dynamic, network-level precision regulation—and in doing so, develop climate-resilient crops that balance stress survival with field productivity.

### 8.4. A Forward-Looking Perspective

The shift we are calling for is not just conceptual. It is technically achievable through four complementary pillars, as described in the previous section. New in vivo ROS biosensors have changed what we can track. They allow for the visualization of subcellular ROS fluctuations at high spatiotemporal resolution, which is speeding up mechanistic studies of redox homeostasis under single and combined stresses [3]. The “dark side of antioxidants” is not that antioxidant proteins or metabolites are intrinsically toxic. It is that the “more-is-better” dogma has kept us from seeing the nuanced, context-dependent logic of plant redox management. What distinguishes this review from earlier work is that we have tried to integrate the signaling, metabolic, and spatiotemporal dimensions of redox regulation to identify where the line falls between beneficial and detrimental antioxidant activity. This is a question that earlier reviews did not systematically address. Moving beyond the oversimplified view requires recognizing ROS as signals, antioxidants as network components, and redox homeostasis as a flexible, condition-dependent balancing act. This will improve our understanding of plant stress physiology and help us develop high-yield, stress-tolerant varieties for a changing climate [10,99].

## Figures and Tables

**Table 1:** Representative experimental cases demonstrating that enhanced or constitutive antioxidant capacity failed to improve, or even impaired, abiotic stress tolerance.

Species	Stress Type	Genetic Manipulation/Observation	Outcome	Reference
*Nicotiana tabacum*	Paraquat, photooxidative stress	Overexpression of GST/GPX	No increase in paraquat tolerance or photooxidative protection	Roxas et al., 1997 [42]
*Gossypium hirsutum*	Salinity, chilling, herbicides	Overexpression of tobacco GST Nt107	No improved tolerance; 50% more GSSG; doubled MDA	Light et al., 2005 [43]
*Citrus sinensis*	In vitro regeneration	Overexpression of PHGPx	Regeneration failure; interference with shoot organogenesis	Faltin et al., 2010 [29]
*Sedum alfredii*	Growth conditions	Overexpression of NfFeSOD	Growth retardation; ~50% reduced H_2_O_2_; impaired ROS signaling	Gao et al., 2016 [44]
*Hordeum vulgare*	Salinity	Constitutive antioxidant levels	Higher AO activity in sensitive variety; no correlation with tolerance	Maksimović et al., 2013 [28]
*Arabidopsis thaliana*	Freezing	Natural variation across accessions	No correlation between AO activity and freezing tolerance	Distelbarth et al., 2018 [45]
*Populus* (poplar)	Osmotic stress	Overexpression of PtoMYB99	Weakened SOD, POD, CAT activity; increased ROS and MDA	Long et al., 2024 [31]
*Salvia miltiorrhiza*	Drought	Natural stress response	Decreased CAT activity; increased H_2_O_2_ and oxidative damage	Zhang et al., 2025 [46]
*Helianthus annuus*	Drought	Natural stress response	CAT activity decreased under drought stress	Ameen et al., 2024 [47]
*Quercus* and *Pinus*	Drought	Natural stress response	APX and CAT activities reduced in most cases	Schwanz et al., 2001 [48]
*Bruguiera parviflora*	Salinity	Natural stress response	CAT activity declined; other AOs enhanced	Parida et al., 2004 [49]
*Cucumis sativus*	Chilling	Natural stress response	CAT activity decreased; SOD, APX, GR enhanced	MacRae and Ferguson, 1985 [50]

**Table 2:** Summary of advanced techniques for studying plant redox regulation.

Technique	Main Application	Key Benefits	Current Limitations
roGFP-based biosensors (roGFP2-PRXIIB)	Real-time H_2_O_2_ tracking in subcellular compartments	High spatiotemporal resolution; in vivo applicability; compartment-specific targeting	Requires specialized equipment; limited to laboratory settings; probe stability concerns
Transcriptomics (RNA-Seq)	Global gene expression profiling under stress	Comprehensive coverage; identifies regulatory networks	Static snapshot; requires validation; high data complexity
Proteomics (LC-MS/MS)	Protein abundance and PTM profiling	Direct functional readout; identifies oxiPTMs	Low abundance protein detection; dynamic range limitations
Metabolomics (GC-MS/LC-MS)	Metabolic pathway analysis	Captures downstream functional outcomes	Metabolite identification challenges; coverage incompleteness
Multi-omics integration	Systems-level network reconstruction	Holistic understanding; identifies cross-layer regulation	Data integration complexity; high costs; cross-study comparability issues
AI/Machine learning platforms	Predictive modeling of redox networks	Accelerates node identification; pattern recognition	Requires large training datasets; black-box interpretability

## Data Availability

No new data were generated in this work.

## Abbreviations

The following abbreviations are used in this manuscript:

ROS	Reactive oxygen species
RNS	reactive nitrogen species
RSS	Reactive sulfur species
SOD	Superoxide dismutase
POD	Peroxidase
CAT	Catalase
APX	Ascorbate peroxidase
GR	Glutathione reductase
MDA	Malondialdehyde
AO	Ascorbate oxidase
GPX	Glutathione peroxidase
GST	Glutathione S-transferase
SAA	Systemic acquired acclimation
NADPH	Nicotinamide adenine dinucleotide phosphate hydrogen
RBOH	Respiratory burst oxidase homolog
PHGPx	Phospholipid hydroperoxide glutathione peroxidase
